# Navigated laparoscopic microwave ablation of tumour mimics in pig livers: a randomized ex-vivo experimental trial

**DOI:** 10.1007/s00464-020-08180-5

**Published:** 2020-12-07

**Authors:** M. N. Thomas, G. Dieplinger, R. R. Datta, R. Kleinert, H. F. Fuchs, A. Bunck, M. Peterhans, C. J. Bruns, D. Stippel, R. Wahba

**Affiliations:** 1 Department of General, Visceral, Cancer and Transplant Surgery, University of Cologne, Faculty of Medicine and University Hospital Cologne, Kerpener Straße 62, 50937 Cologne, Germany; 2 Department of Diagnostic and Interventional Radiology, University of Cologne, Faculty of Medicine and University Hospital Cologne, Cologne, Germany; 3 CAScination AG, Bern, Switzerland

**Keywords:** Microwave ablation, Stereotactic navigation, Liver thermoablation

## Abstract

**Background:**

In order to efficiently perform laparoscopic microwave ablation of liver tumours precise positioning of the ablation probe is mandatory. This study evaluates the precision and ablation accuracy using the innovative laparoscopic stereotactic navigation system CAS-One-SPOT in comparison to 2d ultrasound guided laparoscopic ablation procedures.

**Methods:**

In a pig liver ablation model four surgeons, experienced (*n* = 2) and inexperienced (*n* = 2) in laparoscopic ablation procedures, were randomized for 2d ultrasound guided laparoscopic or stereotactic navigated laparoscopic ablation procedures. Each surgeon performed a total of 20 ablations. Total attempts of needle placements, time from tumor localization till beginning of ablation and ablation accuracy were analyzed.

**Results:**

The use of the laparoscopic stereotactic navigation system led to a significant reduction in total attempts of needle placement. The experienced group of surgeons reduced the mean number of attempts from 2.75 ± 2.291 in the 2d ultrasound guided ablation group to 1.45 ± 1.191 (*p* = 0.0302) attempts in the stereotactic navigation group. Comparable results could be observed in the inexperienced group with a reduction of 2.5 ± 1.50 to 1.15 ± 0.489 (*p* = 0.0005). This was accompanied by a significant time saving from 101.3 ± 112.1 s to 48.75 ± 27.76 s (*p* = 0.0491) in the experienced and 165.5 ± 98.9 s to 66.75 ± 21.96 s (*p* < 0.0001) in the inexperienced surgeon group. The accuracy of the ablation process was hereby not impaired as postinterventional sectioning of the ablation zone revealed.

**Conclusion:**

The use of a stereotactic navigation system for laparoscopic microwave ablation procedures of liver tumors significantly reduces the attempts and time of predicted correct needle placement for novices and experienced surgeons without impairing the accuracy of the ablation procedure.

Radiofrequency and microwave ablations are increasingly used to treat primary and secondary liver malignancies as an alternative to surgery or as an adjunct to surgical resection in patients with impaired liver function, technically non-resectable liver tumours, distinct bilobar tumour manifestation and patients in which large liver resections cannot be safely performed due to concurrent medical conditions [1].

Currently the microwave ablation applicator is placed in a laparoscopic or open procedure under 2D ultrasound guidance into the lesion.

Accurate positioning of the applicator into the lesion is crucial in order (a) to achieve complete ablation of the targeted lesion and (b) to minimize the risk of injury of adjacent structures. This technique is associated with a significant learning curve and explains the reported high local recurrence rates [2].

Tumour targeting and ablation applicator placement is an even greater problem particularly in laparoscopic ablation procedures, since the intuitive spatial correlation of the hand holding the ultrasound probe and the hand placing the applicator in the tumour is displayed on two different screens [3].

In addition, major complications following liver thermoablation including intra-peritoneal bleeding, portal or hepatic vein thrombosis, bile duct injury, liver dysfunction, and intestinal perforation remain a clinically relevant problem in up to 10% of patients [4, 5].

These complications are often directly linked to the trauma associated with applicator placement. Even oncologic outcome could be deteriorated by inadequate applicator placement, as the risk of tumour seeding in the needle tract is linked directly to multiple needle insertions [6]. Therefore, the number of attempts for proper applicator placement should be minimized during the ablation procedure and is of utmost importance. The use of 2D-ultrasound for proper applicator placement in a 3D-space requires a high degree of expertise and skills. There is a significant learning curve in thermoablation of liver tumours. After 50 performed RFA procedures for liver tumours complication rate drops and the rate of complete ablation increases [7]. Especially highly experienced hepatopancreatobiliary surgeons were able to hit a target in a single pass using 2D-ultrasound guidance in as low as 59% of the cases [8].

It could be demonstrated that the use of magnetic and infrared 3D guided ablation techniques led to significantly higher accuracy in applicator placement during simulated ablation procedures [8–10].

The aim of this study was to evaluate targeting precision and ablation accuracy using the innovative laparoscopic stereotactic navigation system CAS-One-SPOT in comparison to 2d ultrasound guided laparoscopic ablation in a randomized experimental ex vivo porcine tumour mimic model (ClinicalTrials.gov: NCT04106453).

## Methods

### SPOT software

**Fig. 1:**
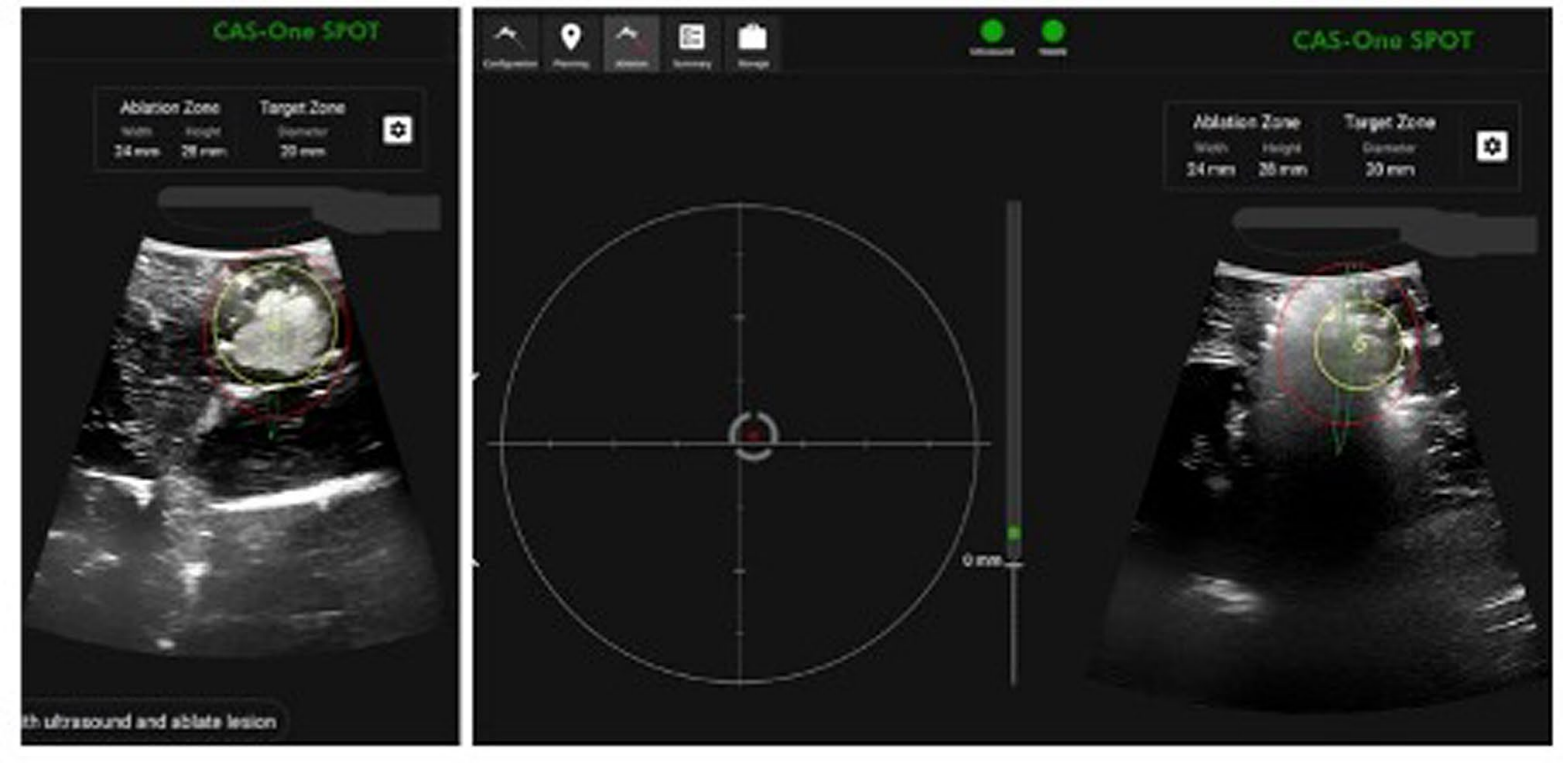
CASone-SPOT system software. Left image showing the located and marked hepatic lesion. Right image showing the navigated puncture of the lesion and the ablation process. Yellow circle showing the middle of the ablation. Red circle showing the estimated margins of the ablation zone (Color figure online)

The CAS-One-SPOT system (CAScination AG, Bern, Switzerland) is an innovative electromagnetic tracking and navigation system for laparoscopic microwave ablation. The device is comprised of a navigation platform which connects to a table-mounted, electromagnetic field generator. The position of a 4-way articulated, laparoscopic ultrasound probe (BK Medical, Herlev, Denmark) is tracked by a dedicated adapter clip including an electromagnetic sensor. In parallel, the position of an ablation needle is tracked using a sensor attached to the needle shaft by means of an adapter clip. The navigation software then calculates the expected intersection point between the needle path and the ultrasound image and displays the position of the needle tip as well as the expected ablation volume. In the first step, the user is asked to define a target point for the ablation needle on the ultrasound image (Fig. 1, left). In the second step, needle guidance towards the target point is provided by means of a bullseye targeting display and a live ultrasound image with position of a simulated ablation zone is displayed (Fig. 1, right). The software documents the target points selected and the positioning of the needle/ablation zone relative to the target (Fig. 1). With the SPOT system, targeting and ablation needle placement is possible from any angle. In contrast, using the conventional laparoscopic technique, the needle placement must be performed in-plane of the ultrasound device.

### Tumour mimics

Tumour mimics were generated in pig livers as described earlier by Scott et al. [11]. Briefly, a gelantine-like mixture of 3% agarose, 3% cellulose, 7% glycerol, and 0.05% methylene blue was prepared. Before injection, the mixture was heated to 70 °C and a constant volume was injected into the pre-cooled (4 °C) pig livers forming intrahepatic tumour lesions of 1–1.5 cm size. No more than 6 tumour mimics were injected into each pig liver. The tumour mimics have been earlier evaluated for radiofrequency ablation in our working group [10].

### Study design

**Fig. 2:**
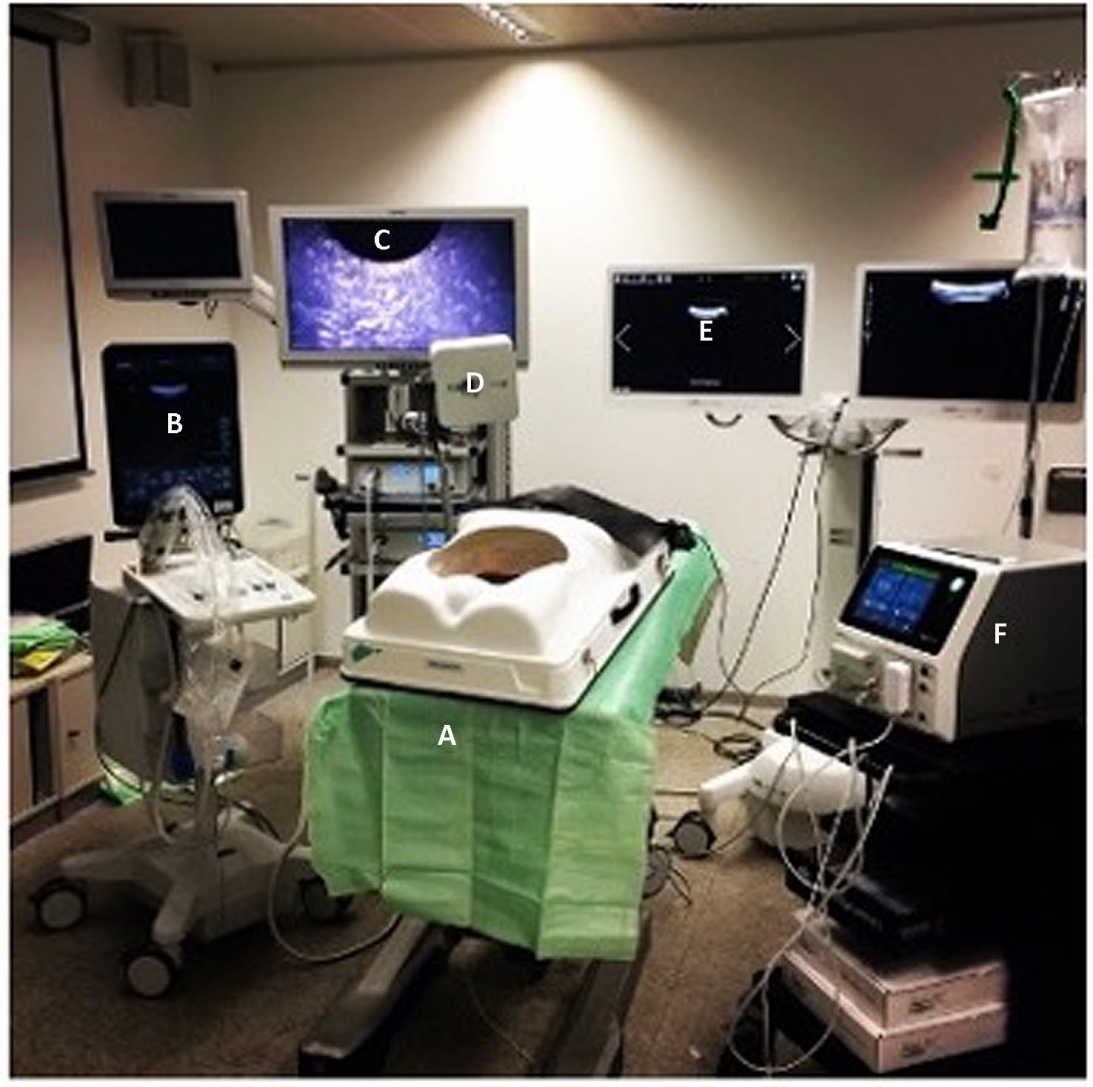
Experimental setup (**A** laparoscopic torso trainer, **B** Ultrasound device, **C** laparoscopic tower, **D** electromagnetic field generator, **E** CAS-One System, **F** Ablation power unit)

After tumour mimic injection the pig livers (weighing about 1500 g) were fixed on a cork tile and placed into a laparoscopic torso trainer. Ports for the laparoscopic camera (Einstein Vision, B.Braun, Germany) and the laparoscopic ultrasound probe (BK Medical, USA) were placed through a foam plate on the top front of the laparoscopic trainer, simulating the rigidity of the abdominal wall (Fig. 2).

This IRB-approved study was designed as a randomized cross-over trial.

**Fig. 3:**
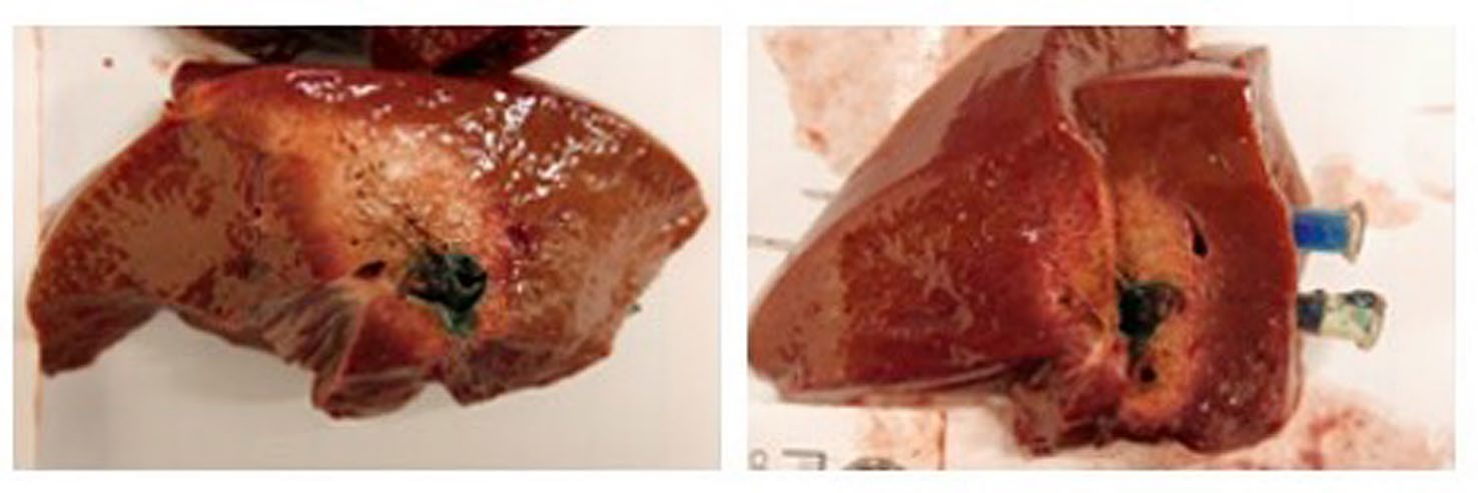
Intrahepatic tumour mimic (blue) and ablation zone (orange) in axial (left) and transverse plane (right) (Color figure online)

**Fig. 4:**
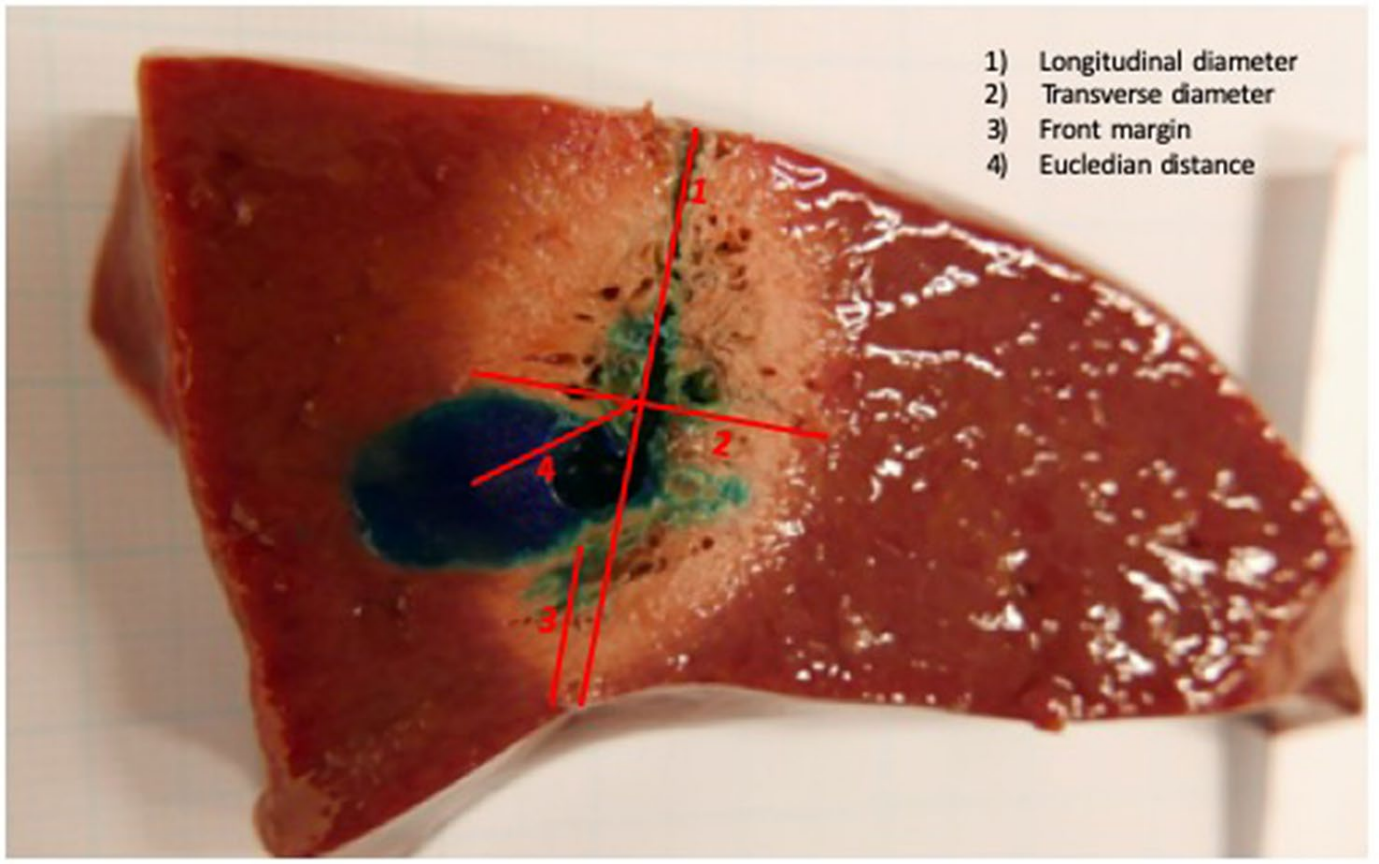
Pig liver specimen exemplary showing the performed measurements using Image-J to determine accuracy of the ablation procedure in axial plane. The ablation zone does not fully cover the tumour mimic (here microwave ablation performed in the conventional 2d ultrasound guided laparoscopic technique by an unexperienced surgeon)

A total of 4 surgeons performed the experiments. They were separated into 2 groups. Group 1 surgeons (*n* = 2) were novice to the ablation technique and group 2 surgeons (*n* = 2) were experienced (over 200 and 1000 ablations in human). Each surgeon performed a total of 20 ablations. After initial ultrasound localisation of the hepatic lesion the ablation process was randomized in 2D ultrasound guided or 3D navigated using the SPOT software. Time was measured from first puncture of the foam plate until beginning of ablation for each targeted lesion. Additionally, the number of attempts for correct needle placement was documented. Ablations were performed using the SOLERO (Angiodynamics, USA) microwave tissue ablation system. In order to keep the ablation zone small enough to see differences in the accuracy of needle placement in the different groups, an ablation power of 100 W for 2 min was chosen. After ablation the localisation of the targeted lesion was visualized on the liver surface in order to differentiate between the multiple intrahepatic lesions. For further analyses the pig livers were taken out of the torso trainer and each ablation zone was cut in an standardized axial and transverse plane as described by Mulier et al. [7]. Then the specimens were placed on millimetre paper, photographed and digitalized for further measurements (Figs. 3, 4).

**Fig. 5:**
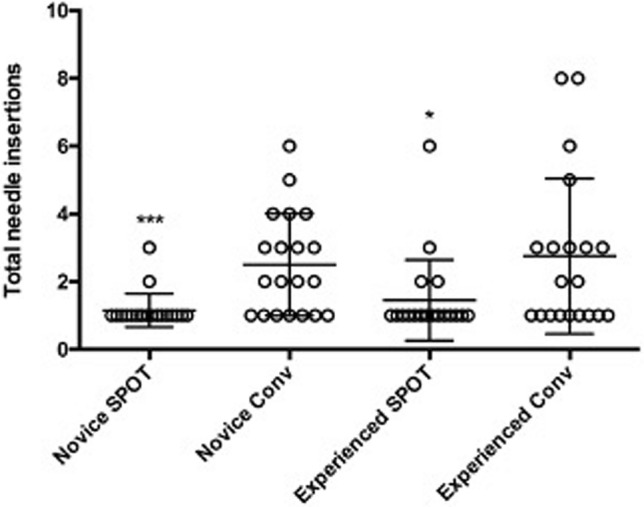
Number of attempts for predicted correct microwave applicator positioning before ablation

Measurements were performed using Image J software v 1.52 following the recommendation of Mulier et al. [7] of a standardized description of coagulation size. Furthermore, the investigator was blinded to the ablation process that was used—SPOT vs. 2d ultrasound guided. In the axial plane the axial diameter, the maximal transverse diameter, the front margin and the Euclidian distance (centre of the ablation zone to centre of the tumour lesion) were measured (Fig. 5). In the transverse plane the maximal transverse diameters and the Euclidian distance were measured. Measurements were performed with the open source software Image J v 1.52. The study was registered at clininicaltrial.gov (NCT04106453).

### Statistical analyses

Statistical analyses were performed using IBM SPSS 23. Graph Pad Prism 6 was used to create figures. Data distributions were assumed to be Gaussian and summarised as mean ± standard deviation and compared by two-sided (Student’s) *t*-tests for paired or for unpaired data.

## Results

### Number of targeting attempts

**Fig. 6:**
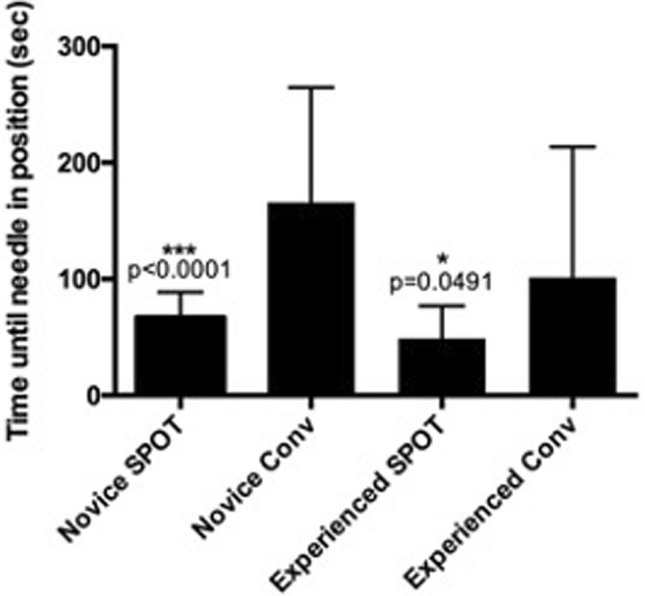
Time from first attempt to puncture the target lesion until the beginning of the microwave ablation

The mean number of targeting attempts until predicted correct positioning of the needle by the inexperienced surgeons using the standard ultrasound 2D guided technique was 2.5 ± 1.5 attempts. With the use of the stereotactic navigation system the mean number of attempts could be reduced to 1.15 ± 0.48 attempts (*p* = 0.0005). The experienced group of surgeons needed a mean of 2.75 ± 2.29 attempts in the conventional ablation setting and were able to reduce the number of attempts to a mean of 1.45 ± 1.19 in the stereotactic navigated ablation group (Fig. 6) (*p* = 0.0302).

### Time until ablation

The inexperienced surgeons needed a mean time of 165.5 ± 98.9 s in the 2d ultrasound guided ablation group from first introduction of the needle until predicted correct positioning of the needle. By using the stereotactic navigation system this could be significantly reduced to a mean of 66.75 ± 21.96 s (*p* < 0.0001).

In line, the experienced surgeons were able to reduce the mean time from first needle puncture to beginning of ablation from a mean of 101.3 ± 112.1 s in the 2d guided ultrasound group to a mean of 48.75 ± 27.76 s in the stereotactic navigated group (p = 0.0491) (Fig. 6).

### Targeting accuracy

Volumetric measurements of the ablation zones revealed no statistically significant difference between the groups (2d ultrasound guided inexperienced vs. stereotactic guidance inexperienced 4.9 ± 0.52cm^2^ vs. 4.33 ± 0.41cm^2^/2d ultrasound guided experienced vs. stereotactic guidance experienced 5.27 ± 0.68cm^2^ vs. 7.17 ± 0.83cm^2^). Additionally the front margin (end of tumour to end of ablation) (2d ultrasound guided inexperienced vs. stereotactic guidance inexperienced 0.09 cm ± 0.69 vs. 0.22 ± 0.61 (*p* = 0.543)/2d ultrasound guided experienced vs. stereotactic guidance experienced − 0.10 cm ± 0.51 vs. − 0.06 cm ± 0.50 (*p* = 0.838)) nor the Eucledian-distance (2d ultrasound guided inexperienced vs. stereotactic guidance inexperienced 0.95 cm ± 0.46 vs. 0.89 ± 0.62 (*p* = 0.73)/2d ultrasound guided experienced vs. stereotactic guidance experienced 1.06 cm ± 0.41 vs. 0.92 cm ± 0.30 (*p* = 0.228)) revealed no statistically significant measurements between the analysed groups.

## Discussion

Fast and accurate placement of the ablation electrode is crucial in laparoscopic microwave ablation for malignant liver tumours to reduce surgical complications and provide optimal oncologic outcome. Using the conventional 2D ultrasound guided technique a relevant learning curve has to be passed through. Targeting systems seem to help surgeons here. In general, using the system is complex. Our study demonstrates that the use of an electromagnetic 3D guided ultrasounds navigation system in laparoscopic microwave ablation procedures leads to a significant reduction in attempts until predicted correct needle placement is accomplished. In our experimental setting the use of the 3D guided ultrasound ablation method led to a significant reduction in needle withdrawals of 49.5% attempts. This reduction was accompanied by a significant time saving of 43% from localisation of the intrahepatic target to the beginning of ablation. Even the group of experienced surgeons had high benefit from the SPOT system compared to the conventional ultrasound technique. Needle placement becomes fast and easy. Furthermore, the targeting accuracy did not significantly differ. Although experienced surgeon were faster in the placement of the applicator, interestingly both groups of surgeons, experienced and inexperienced surgeons, had comparable results regarding total attempts of needle placement and accuracy of the ablation procedure even in the conventional ultrasound guided ablation setting. This can in part be explained by the used model of porcine livers. Even if this model is widely accepted for the studying and learning of thermal ablation techniques [10, 11], porcine livers are relatively flat and tumour mimics are predominantly injected near to the liver surface. Therefore, difficult angels and long trajectories through healthy liver parenchyma in order to hit the lesion are rare. The experimental use of bovine livers could increase the degree of difficulty and possibly reveal more differences between the two groups.

Thermal ablation of liver tumours has evolved to be a broadly accepted tool in the management of increasingly complex oncologic patients. However, this procedure is still afflicted with the occurrence of major complications [4, 10].

One feared complication of open, laparoscopic and percutaneous ablation procedures is the release of neoplastic cells along the needle track. This complication occurs in up to 2.5% [6, 12, 13]. The risk of this complication is directly linked to the need of multiple needle placements until correct positioning of the needle is achieved in the centre of the tumour [6, 14]. With the use of the 3d navigated ablation procedure this complication could be possibly diminished due to the significantly reduced number of needle insertions.

Krasnick et al. [9] could demonstrate comparable results in an in vivo porcine model of 3D electromagnetic guided ultrasound ablations. In the used porcine model, a fiducial marker was placed in the liver and kidney and ablation was performed either using the 3D guided ultrasound technique or the conventional 2D guided ultrasound technique. Ablation procedures were conducted by 3 general surgeons experienced in ablation procedures (> 48 ablations performed in clinical routine). In their experimental laparoscopic setup, the use of the electromagnetic guided 3D ultrasound ablation technology led to a significant reduction of 62% in needle placement attempts until the ablation procedure could be conducted as compared to the conventional 2D guided ultrasound ablation. The targeting accuracy, as defined by locating the fiducial marker in the ablation zone, was not significantly different in the analysed groups. The specimen analyses in order to study the accuracy of the performed ablation procedure was carried out in a single plane. However, as the ablation zone is a 3D-elipsoid structure a second sectional plane (transversal and axial) should have been analysed in order to reliably study targeting accuracy. In our experimental setting the tumour mimic localisation was analysed in relation to the ablation zone in 2 sectional planes. We could demonstrate that the use of the 3D guided electromagnetic field in laparoscopic liver ablation was not associated with a diminished targeting accuracy.

Paolucci et al. analysed an experimental electromagnetic ultrasound-based navigation technique for experimental laparoscopic ablation of liver tumor mimics. They could show with an experimental electromagnetic ultrasound-based navigation technique a significant reduction in time for positioning of the ablation probe and a significant reduction in needed repositioning attempts of the ablation needle [15]. However no real ablation of a tumour was performed in this study: only the adequate positioning of the needle was calculated based on a 3D ultrasound image.

Another recently published study by Tinguely et al. analysed the clinical use of laparoscopic image-guided microwave ablation technique for malignant liver tumours [16]. In their retrospective study ablation procedures were performed using an intraoperative rigid four-landmark-based registration technique from a pre-operatively reconstructed 3d model of the liver. This technique however is accompanied with a high degree of technical and financial effort as 3d model reconstruction of the liver is mandatory for every patient before the planed ablation procedure.

The laparoscopic use of the CASOne-Spot System in liver ablations procedures allows precise targeting of liver lesions and is linked to a significant reduction in necessary needle insertions. With the use of this new technology complications observed in conventional 2D guided laparoscopic ablation procedures can be potentially minimized and wider indications for laparoscopic ablation can be achieved.

## Conclusion

The use of an electromagnetic-navigated 3D ultrasound ablation technique in laparoscopic ablation procedures, when compared to the 2D ultrasound guidance, leads to a significant reduction in necessary attempts of needle placement without restricting ablation accuracy. With the clinical use of this new technique laparoscopic microwave ablation could be simplified, thus wider indications for laparoscopic ablations can be developed and complications associated with needle placement could be diminished.
